# A machine learning analysis of difficulty scoring systems for laparoscopic liver surgery

**DOI:** 10.1007/s00464-022-09322-7

**Published:** 2022-05-23

**Authors:** Andrea Ruzzenente, Fabio Bagante, Edoardo Poletto, Tommaso Campagnaro, Simone Conci, Mario De Bellis, Corrado Pedrazzani, Alfredo Guglielmi

**Affiliations:** Department of Surgery, Dentistry, Gynecology and Pediatrics, Division of General and Hepato-Biliary Surgery, University of Verona, P. le L.A. Scuro, 37134 Verona, Italy

**Keywords:** Difficulty scoring system, Laparoscopic liver resection, Patient selection, Machine learning, Textbook outcome

## Abstract

**Introduction:**

In the last decade, several difficulty scoring systems (DSS) have been proposed to predict technical difficulty in laparoscopic liver resections (LLR). The present study aimed to investigate the ability of four DSS for LLR to predict operative, short-term, and textbook outcomes.

**Methods:**

Patients who underwent LLR at a single tertiary referral center from January 2014 to June 2020 were included in the present study. Four DSS for LLR (Halls, Hasegawa, Kawaguchi, and Iwate) were investigated to test their ability to predict operative and postoperative complications. Machine learning algorithms were used to identify the most important DSS associated with operative and short-term outcomes.

**Results:**

A total of 346 patients were included in the analysis, 28 (8.1%) patients were converted to open surgery. A total of 13 patients (3.7%) had severe (Clavien–Dindo ≥ 3) complications; the incidence of prolonged length of stay (> 5 days) was 39.3% (*n* = 136). No patients died within 90 days after the surgery. According to Halls, Hasegawa, Kawaguchi, and Iwate scores, 65 (18.8%), 59 (17.1%), 57 (16.5%), and 112 (32.4%) patients underwent high difficulty LLR, respectively.

In accordance with a random forest algorithm, the Kawaguchi DSS predicted prolonged length of stay, high blood loss, and conversions and was the best performing DSS in predicting postoperative outcomes. Iwate DSS was the most important variable associated with operative time, while Halls score was the most important DSS predicting textbook outcomes. No one of the DSS investigated was associated with the occurrence of complication.

**Conclusions:**

According to our results DDS are significantly related to surgical complexity and short-term outcomes, Kawaguchi and Iwate DSS showed the best performance in predicting operative outcomes, while Halls score was the most important variable in predicting textbook outcome. Interestingly, none of the DSS showed any correlation with or importance in predicting overall and severe postoperative complications.

**Graphical abstract:**

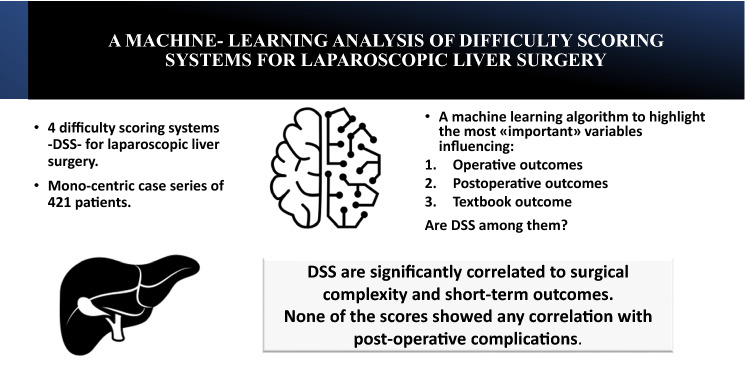

In the last decades, laparoscopic liver resections (LLR) has been worldwide widely adopted and progressively surgeons who initially developed and refined the technique (the so-called “pioneers”) have mentored a new generation of “early adopters,” that uptake LLR earlier and with steeper learning curves [1].

However, the level of complexity of the single procedure must be taken in consideration when approaching LLR, to properly consider each step of the learning curve and also to select patients who could most benefit from a minimally invasive approach.

Various aspects are known to affect the degree of LLR complexity, including patient-related factors (such as body mass index), tumor-related factors (such as size or histology), and surgery-related factors (such as the extent and type of planned resection) [2, 3]. As indicated during the Consensus Conference held in Morioka in 2014, the use of difficulty scoring systems (DSS), that combine all these factors, is strongly recommended to appropriately select patients according to the surgeon’s skill level [4].

DSS have been created and validated for single outcomes, deemed to be associated with increased surgical difficulty, such as blood losses, operative time, and others. In the last years, however, the use of composite measures has been proposed since they are a good indicator of the quality of surgical care. Textbook Outcome (TO) is a composite measure created by aggregating various peri-operative outcomes believed to contribute to optimal results following surgery, with an “all-or-none” approach [5, 6], meaning TO is achieved if every outcome included is achieved.

In the present study, the four most commonly applied DSS (Halls [7], Hasegawa [8], Kawaguchi [9], and Iwate-as remodeled after the 2nd International Consensus Conference [4]) have been evaluated in consecutive patients that underwent LLR at a single Western Institution to evaluate their ability to predict operative, postoperative, and textbook outcomes.

## Methods

All patients who underwent LLR at our institution, both for benign and malignant disease, from January 2014 to December 2020, were included in the study. Data concerning patient demographic features, past medical history, disease characteristics, type of LLR and technical aspects, hospital stay, and 90 days follow-up were obtained from a prospectively maintained database involving all patients who underwent LLR at our institution.

The study was approved by the ethics committee of our institution.

Inclusion criteria were age > 18 years old and a liver resection performed with a laparoscopic approach. Exclusion criteria were lack of data needed to compute one or more of the scores and follow-up shorter than 90 days. Laparoscopic cyst fenestrations were not considered liver resections and therefore were not included in the database. Resections were performed by three surgeons at different stages of the learning curve.

The Brisbane terminology was used to define the extent of the resection and a major hepatectomy was defined as the resection of at least three contiguous segments [10].

Intraoperative events were classified according to the modified Satava classification [11].

Complications were evaluated both within the hospital stay and within 90 days from surgery. They have been classified according to the Clavien–Dindo score [12] and those with a Clavien–Dindo score equal to or greater than 3 were considered as severe complications. In particular, among complications, postoperative liver failure and bile leakage were defined, according to the International Study Group of Liver Surgery classifications, as any alteration of the international normalized ratio (INR) and bilirubin serum levels after the 5th postoperative day and secretion of fluid with an increased bilirubin concentration from intra-abdominal drains on the 3rd postoperative day or the need for radiologic or surgical re-intervention for biliary collections or bile peritonitis, respectively [13, 14].

The operative and postoperative outcomes collected and analyzed were operative time, blood losses and unplanned conversion, length of hospital stay, and postoperative overall and severe (Clavien–Dindo ≥ 3) complications. Three composite outcomes were subsequently used: an “operative outcome,” a “postoperative outcome,” and textbook outcome.

We defined operative outcome (OO) as the absence of conversion to open surgery, operative time ≤ 240 min, and blood losses ≤ 500 ml, while postoperative outcome (PO) as the absence of any complication and a length of hospital stay ≤ 5 days. Finally, textbook outcome (TO) was defined as no moderate/severe (Satava > I) intraoperative events, no severe (Clavien–Dindo > II) complications, no prolonged length of stay (> 75° percentile of the series), radical resection (R0), and no 90 days of hospital readmission and mortality.

### Surgical technique

During surgery, laparoscopic intraoperative ultrasound was routinely performed to confirm the preoperative diagnosis and to evaluate the relationship between the lesions, blood vessels, and bile ducts. Laparoscopic intermittent Pringle’s maneuver was routinely performed and hilar clamping was used if needed to control bleeding and help liver transection, in appropriate cases, selective clamping was performed.

Accurate liver parenchyma dissection was performed utilizing an ultrasonic surgical aspiration system with selective isolation of intraparenchymal vessels, laparoscopic radiofrequency or harmonic scalpel was selectively applied as appropriate, major vessels were selectively sealed with endo-clips or vascular staplers.

### Difficulty score calculation

For every patient eligible for this study, each one of the four scores was calculated, based on the clinical, radiological, and operative data. When multiple resections were performed during a single procedure, scores were calculated on the most challenging one.

Halls score, also mentioned as Southampton DSS, was developed to predict intraoperative complications, graded with the Satava classification system. It is based on the type of resection (4 points for major resections, 2 points for anatomical resections of one or two segments including posterosuperior segments, 0 points to every other resection), tumor size (3–5 cm: 2 points, more than 5 cm: 3 points), type of the tumor (2 points if malignant), previous open liver resection (5 points), or neoadjuvant chemotherapy (1 point). According to this DSS, the risk of intraoperative complication is classified as low (less than 2), moderate (3 to 5), high (6 to 9), and extremely high (10 to 15) [7].

Hasegawa score was developed for predicting operative time and the resulting model was evaluated for the operative outcomes (blood losses, conversion, complications, and length of stay). The variables considered are the type of LLR (wedge resections and left lateral sectionectomy 0 points, segmentectomy 2 points, major resections 3 points), tumor location (segments 7–8: 2 points, segment 5–6: 1 point, segment 2–3–4: 0 points), BMI (≥ 30 kg/m^2^ 1 point), and platelet count (≤ 100 × 10^9^/L 1 point). The surgical difficulty was classified into three levels: low-difficulty procedure, score ≤ 1; medium difficulty procedure, score 2–3; and high difficulty procedure, score ≥ 4 [8].

Kawaguchi score, also known as Difficulty of LLR classification or as IMM (Institute Mutualiste Montsouris), was developed to predict 90-day postoperative morbidity and mortality. According to this score patients are divided into three groups of increasing difficulty, Group I includes wedge resections and left lateral sectionectomy, Group II includes left hepatectomy and anterolateral anatomical segmentectomy, and Group III that includes posterosuperior segmentectomy, right hepatectomy, central hepatectomy, and right- or left-extended hepatectomy [9].

Ban’s score was the first one developed and was later updated during the Morioka consensus conference. The updated version of this DSS (known as Iwate score) was used in this study. Patients are divided into four difficulty groups: low (1–3 points), intermediate (4–6 points), advanced (7–9 points), and expert (10–12 points). The following factors are considered in the computation: tumor size (≥ 3 cm, 1 point), tumor location (1 to 3 points for anterolateral segments, 4 to 5 points for posterosuperior segments), the extent of liver resection (0 points for wedge resections, 2 for left lateral sectionectomy, 3 points for segmentectomy, 4 points for sectionectomy and more), proximity to major vessels (1 point), liver function (Child–Pugh B, 1 point), and the use of hybrid/hand-assisted technique (− 1 point) [4].

### Statistical analysis

Statistical analysis was conducted in R (R Core Team, 2014) and figures were produced using the package ggplot2 (Wickham, 2009). To easily compare both scores with three difficulty categories (Hasegawa and Kawaguchi) and four difficulty categories (Iwate and Halls), the two highest risk categories of the latter ones have been considered as one. The degree of correlation among the various scores was tested using Spearman’s ranked correlation coefficient. Five variables have been used as indicators of operative outcomes (blood losses, operative time, conversion) and short-term outcomes (complications and length of hospital stay), and three composite measures have been created: operative outcome (including blood losses, operative time, and conversion), postoperative outcome (including length of stay and complications), and textbook outcome, defined as no moderate/severe (Satava > I) intraoperative events, no severe (Clavien Dindo > II) complications, no prolonged length of stay (> 75° percentile of the series), radical resection (R0), no 90 days of hospital readmission and mortality. To explore how each DSS performs in predicting operative and short-term outcomes, a logistic regression analysis has been conducted, to evaluate if increasing DSS difficulty classes was associated with worse operative and short-term outcomes, and with composite outcomes. In addition, random survival models (random forest models) were used to identify the most important DSS associated with operative, short-term, and composite outcomes. Random forest model is a machine learning algorithm that uses bootstrap aggregation of single decisional trees. In simpler words, a random forest model combines many individual decisional trees, that are individually inaccurate, in a single model that gives the most accurate previsions.

When applied, as in this case, to a regression problem, it can classify the most important variables in predicting a determined outcome, based on how many decisional trees return said variable [15].

## Results

### Population characteristics, operative and short-term outcomes, composite outcomes

**Fig. 1:**
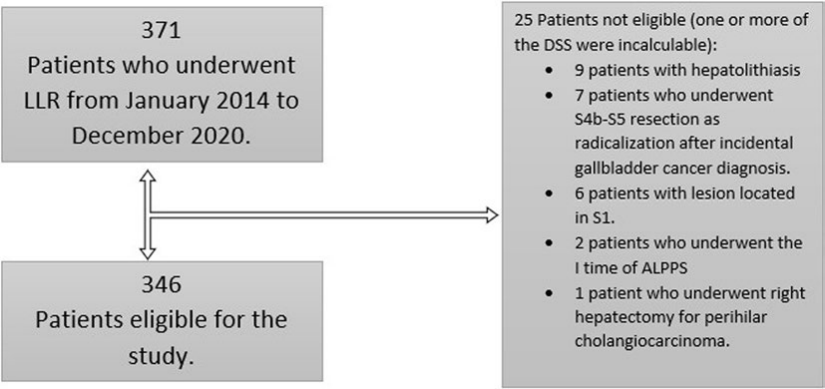
Flowchart showing patient selection for this study

**Fig. 2:**
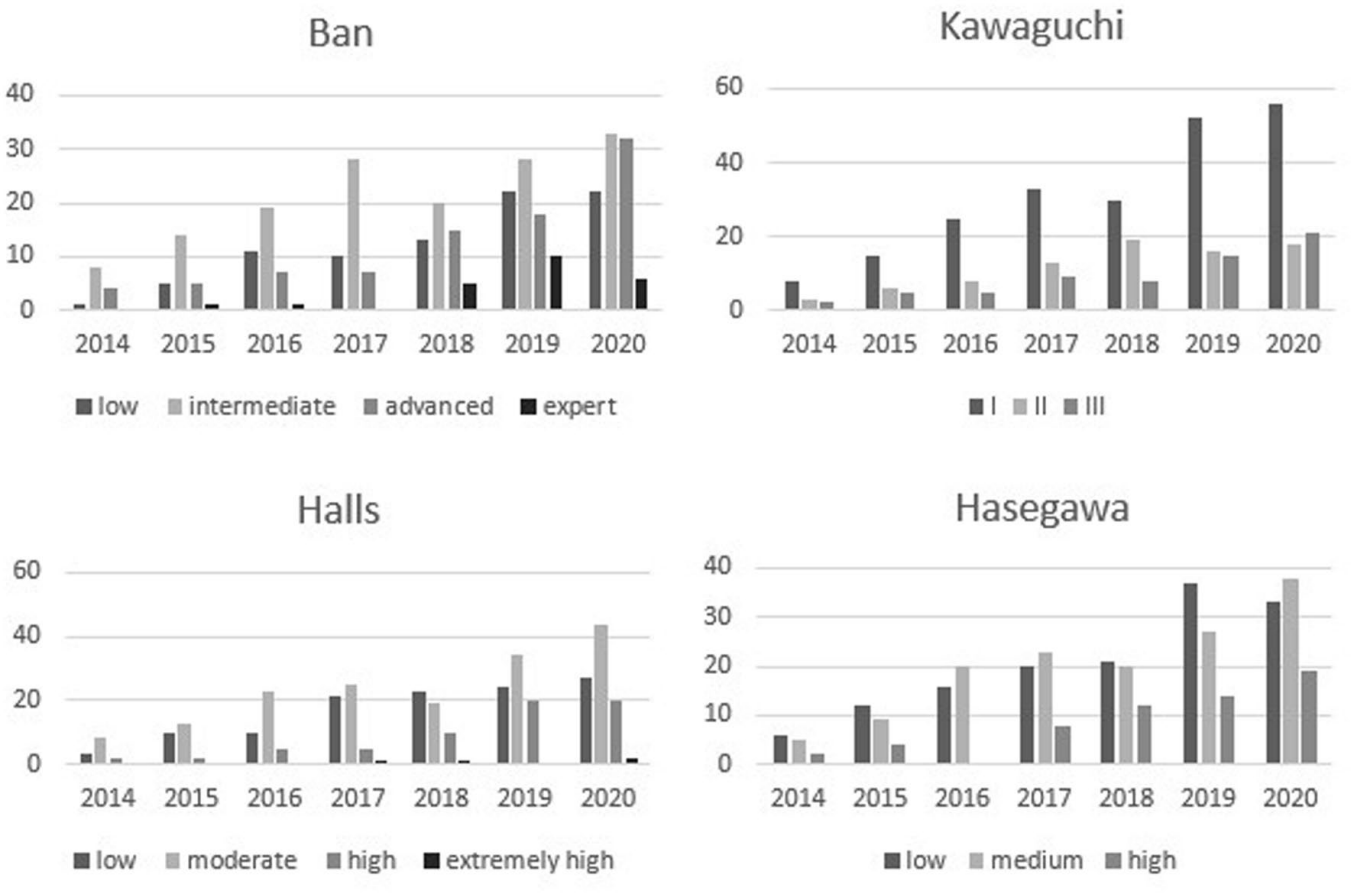
Increasing number of laparoscopic liver resection, and the difficulty of the procedures, performed from 2014 to 2020

A total of 371 patients underwent LLR at our institution during the considered study period, but after the application of exclusion criteria 346 patients were eligible for the study, as described in Fig. 1; Fig. 2 shows the number of LLR performed and the distribution according to the DSS in the study period.

The characteristics of the analyzed population are summarized in Table 1. Most patients were male (201; 58.1%), age was ≥ 65 years in 186 (53.7%) cases, and BMI was ≥ 25 in 195 (56.4%) cases. At the histopathological examination, 163 (47.1%) patients had healthy liver, 64 (18.5%) showed signs of steatosis, and 119 (34.4%) of cirrhosis; 177 patients (96.7%) showed preserved liver function (Child–Pugh A) and 63 patients (18.2%) showed signs of portal hypertension. The anaesthesiologic risk was high (ASA 3–4) in 32.9% of the patients.

**Table 1:** Baseline features of MILS patients (*N* = 346)

Variables	*N* (%)
Age	
< 65 years	160 (46.3)
≥ 65 years	186 (53.7)
Gender	
Male	201 (58.1)
Female	145 (41.9)
BMI	
< 25	151 (43.6)
≥ 25	195 (56.4)
ASA score	
1–2	232 (67.1)
3–4	114 (32.9)
Cardiologic comorbidities	
No	295 (85.3)
Yes	51 (14.7)
Vascular comorbidities^a^	
No	163 (47.1)
Yes	183 (52.9)
Diabetes	
No	272 (78.6)
Yes	74 (21.4)
Respiratory comorbidities	
No	317(91.6)
Yes	29(8.4)
Neurologic comorbidities	
No	320 (94.2)
Yes	20 (5.8)
Chronic kidney disease	
No	296 (85.5)
Yes	50 (14.5)
Liver histology	
Healthy	163 (47.1)
Steatosis	64 (18.5)
Cirrhosis	119 (34.4)
Portal vein hypertension^b^	
No	283 (81.8)
Yes	63 (18.2)
Platelets (× mm^3^)	
≤ 100,000	44 (12.7)
> 100,000	302 (87.3)
Pre-operative chemotherapy	
No	45 (13)
Yes	301 (87)
Disease	
HCC	151 (43.6)
CRLM	64 (18.4)
NCRLM	29 (8.4)
CCC	42 (12.2)
Benign	60 (17.4)
Operative time	
≤ 240 min	178 (51.4)
> 240 min	168 (48.6)
Blood loss	
≤ 500 ml	322 (93.1)
> 500 ml	24 (6.9)
Conversion	
No	318 (91.9)
Yes	28 (8.1)
Transfusions	
No	325 (93.9)
Yes	21 (6.1)
Intraoperative events^c^	
0	305 (88.1)
I	13 (3.8)
II	28 (8.1)
III	0 (0)
Complications	
No	251 (72.7)
Clavien–Dindo 1–2	82 (23.6)
Clavien–Dindo ≥ 3	13 (3.7)
Length of stay	
≤ 5 days	210 (60.7)
> 5 days	136 (39.3)
90 days of mortality	0 (0)
90 days of readmission rate	4 (1.2)
Radicality of the resection	
R0	312 (90.2)
R1	34 (9.8)
Halls	
Low risk	118 (34.1)
Moderate	163 (47.1)
High/extremely high	65 (18.8)
Hasegawa	
Low	145 (41.9)
Medium	142 (41)
High	59 (17.1)
Kawaguchi	
I	215 (62.1)
II	74 (21.4)
III	57 (16.5)
IWATE	
Low	84 (24.2)
Intermediate	150 (43.4)
Advanced/expert	112 (32.4)
Operative outcome	
Yes	166 (48)
No	180 (52)
Postoperative outcome	
Yes	208 (60.1)
No	138 (39.9)
Textbook outcome	
Yes	228 (66.1)
No	117 (33.9)

^a^Including Arterial Hypertension

^b^Portal Vein Hypertension assessed by platelet count, spleen dimension, presence of esophageal varices, or collateral circulations

^c^Classified according to Modified Satava Classification [11]

LLR was performed for malignant disease in 82.6% of the patients: 151 (43.6%) for hepatocarcinoma, 64 (18.4%) for colo-rectal liver metastasis, 29 (8.4%) for non-colo-rectal liver metastasis, and 42 (12.2%) for biliary cancers.

The operative time was longer than 240 min in 168 cases (48.6%), 21 (6.1%) patients received blood transfusions, and 24 patients (6.9%) had blood losses greater than 500 ml. In 28 cases (8.1%) unplanned conversion was needed; reasons of conversion to open surgery were the extent of the tumor in 19 cases, technical reasons (mostly adhesions) in 7 cases, and uncontrollable hemorrhage in 2 cases. According to Satava classification mild (grade I), intraoperative events have been registered in 13 patients (3.8%), while moderate events (grade II) in 28 patients (8.1%); no severe (grade III) events were registered.

There was no 90 days of mortality in the study population, but 4 patients (1.2%) were re-admitted within 90 days from surgery. The overall complication rate was 27.3%, whereas severe complication rate was 3.7%; the median value of the length of hospital stay was 5 days, a longer stay was observed in 136 cases (39.3%). A radical (R0) resection was obtained in 90.2% of the patients.

After combining the outcomes in composite measures, we found that operative outcome (OO) was reached in 166 (48%) patients, postoperative outcome (PO) in 208 (60.1%) patients, and textbook outcome (TO) in 228 (66.1%) patients.

The distribution of the patients based on the difficulty scores was as follows: Kawaguchi group I in 215 (62.1%), group II in 74 (21.4%), group III in 57 (16.5%); Halls low risk in 118 (34.1%), moderate risk in 163 (47.1%), high/extremely high risk in 65 (18.8%); Hasegawa low difficulty in 145 (41.9%), medium difficulty in 142 (41%), high difficulty in 59 (17.1%); Iwate low difficulty in 84 (24.2%), intermediate difficulty in 150 (43.4%), advanced/expert difficulty in 112 (32.4%).

**Fig. 3:**
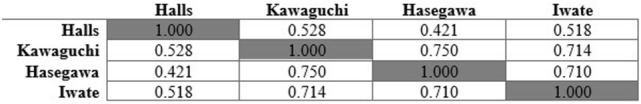
Linear correlation between the DSS measured with the Spearman correlation coefficient

The correlation among the different DSS was investigated using Spearman’s ranked correlation coefficient; a strong relationship was found among Kawaguchi and Hasegawa scores (*ρ* = 0.75), while the weakest relationship was found among Halls and Hasegawa scores (*ρ* = 0.421) as shown in Fig. 3.

### Increasing difficulty according to DSS and operative and short-term outcomes

A logistic regression model was applied to operative and short-term outcomes and composite outcomes to investigate the relationship with DSS classes; results are shown in Table 2.

**Table 2:** Logistic regression for the different DFS tested against the chosen variables

Difficulty score tested	OR	CI 95%	*p* value
*Operative time* (≥ 240 min)			
Kawaguchi			
I	–	–	*–*
II	3.24	[1.88–5.69]	< *0.001*
III	7.8	[3.94–16.68]	< *0.001*
Halls			
Low risk	–	–	*–*
Moderate risk	2.85	[1.73–4.79]	< *0.001*
High/extremely high risk	10.75	[5.31–23.08]	< *0.001*
Hasegawa			
Low risk	–	–	*–*
Medium risk	4.07	[2.49–6.77]	< *0.001*
High risk	9.96	[4.98–21.12]	< *0.001*
IWATE			
Low	–	–	*–*
Intermediate	2.41	[1.33–4.50]	*0.004*
Advanced/expert	11.9	[6.16–23.99]	< *0.001*
*Blood loss* (≥ 500 ml)			
Kawaguchi			
I	–	–	*–*
II	2.14	[0.75–5.79]	0.13
III	2.86	[0.99–7.84]	*0.04*
Halls			
Low risk	–	–	*–*
Moderate risk	12.7	[2.54–231.52]	*0.01*
High/extremely high risk	14.1	[2.43–267.19]	*0.01*
Hasegawa			
Low risk	–	–	*–*
Medium risk	4.77	[1.49–21.14]	*0.01*
High risk	7.42	[2.06–34.89]	*0.003*
IWATE			
Low	–	–	*–*
Intermediate	1.12	[0.28–5.44]	0.87
Advanced/expert	4.17	[1.32–18.47]	*0.02*
*Need for unplanned conversion*			
Kawaguchi			
I	–	–	*–*
II	4.48	[1.71–11.32]	*0.001*
III	9.32	[1.09–9.01]	*0.04*
Halls			
Low risk	–	–	*–*
Moderate risk	12.73	[2.54–231.52]	*0.01*
High/extremely high risk	14.12	[2.43–267.19]	*0.005*
Hasegawa			
Low risk	–	–	*–*
Medium risk	1.87	[0.77–4.82]	0.17
High risk	1.93	[0.61–5.83]	0.24
IWATE			
Low	–	–	*–*
Intermediate	2.34	[0.72–10.52]	0.19
Advanced/expert	3.54	[1.09–15.84]	0.054
*Length of hospital stay* (≥ 5 days)			
Kawaguchi			
I	–	–	*–*
II	2.07	[1.20–3.56]	*0.008*
III	4.37	[2.36–8.32]	< *0.001*
Halls			
Low risk	–	–	*–*
Moderate risk	1.19	[0.72–1.97]	0.48
High/extremely high risk	3.15	[1.69–5.98]	< *0.001*
Hasegawa			
Low risk	–	–	*–*
Medium risk	1.64	[1.01–2.69]	*0.04*
High risk	4.12	[2.19–7.90]	< *0.001*
IWATE			
Low	–	–	*–*
Intermediate	1.36	[0.76–2.48]	0.29
Advanced/expert	5.28	[1.81–6.11]	< *0.001*
*Overall complications*			
Kawaguchi			
I	–	–	*–*
II	1.34	[0.75–2.40]	0.31
III	1.37	[0.72–2.60]	0.32
Halls			
Low risk	–	–	*–*
Moderate risk	1.43	[0.82–2.48]	0.2
High/extremely high risk	1.81	[0.92–3.55]	0.08
Hasegawa			
Low risk	–	–	*–*
Medium risk	1.38	[0.81–2.35]	0.23
High risk	2.25	[1.17–4.33]	*0.01*
IWATE			
Low	–	–	*–*
Intermediate	0.83	[0.45–1.53]	0.56
Advanced/expert	1.25	[0.67–2.34]	0.47
*Severe complications* (*CD* ≥ 3)			
Kawaguchi			
I	–	–	*–*
II	3.01	[0.73–12.37]	0.12
III	5.07	[1.31–19.55]	*0.02*
Halls			
Low risk	–	–	*–*
Moderate risk	2.6	[0.53–12.75]	0.23
High/extremely high risk	3.8	[0.67–21.35]	0.12
Hasegawa			
Low risk	–	–	*–*
Medium risk	6.35	[0.75–53.45]	0.08
High risk	16.3	[1.91–138.59]	*0.01*
IWATE			
Low	–	–	*–*
Intermediate	8.61	[NA–NA]	0.98
Advanced/expert	2.41	[NA–NA]	0.98
*Failure of operative outcome*			
Kawaguchi			
I	–	–	–
II	3.58	[2.04–6.28]	< *0.001*
III	14.63	[6.00–35.64]	< *0.001*
Halls			
Low risk	–	–	–
Moderate risk	2.85	[1.72–4.70]	< *0.001*
High/extremely high risk	14.75	[6.58–33.07]	< *0.001*
Hasegawa			
Low risk	–	–	–
Medium risk	4.11	[2.51–6.75]	< *0.001*
High risk	12.01	[5.57–25.92]	< *0.001*
IWATE			
Low	–	–	–
Intermediate	2.58	[1.42–4.69]	*0.002*
Advanced/expert	15.66	[7.74–31.66]	< *0.001*
*Failure of postoperative outcome*			
Kawaguchi			
I	–	–	–
II	2.02	[1.18–3.47]	*0.01*
III	4.17	[2.25–7.73]	< *0.001*
Halls			
Low risk	–	–	–
Moderate risk	1.22	[0.74–2.02]	0.42
High/extremely high risk	3.36	[1.79–6.63]	< *0.001*
Hasegawa			
Low risk	–	–	–
Medium risk	1.69	[1.03–2.76]	*0.03*
High risk	4.43	[2.33–8.43]	< *0.001*
IWATE			
Low	–	–	–
Intermediate	1.4	[0.78–2.52]	0.25
Advanced/expert	3.41	[1.85–6.26]	< *0.001*
*Failure of textbook outcome*			
Kawaguchi			
I	–	–	–
II	1.98	[1.14–3.45]	*0.01*
III	2.85	[1.56–5.20]	< *0.001*
Halls			
Low risk	–	–	–
Moderate risk	1.58	[0.93–2.69]	0.08
High/extremely high risk	2.94	[1.54–5.59]	< *0.001*
Hasegawa			
Low risk	–	–	–
Medium risk	1.57	[0.94–2.61]	0.08
High risk	2.99	[1.58–5.63]	< *0.001*
IWATE			
Low	–	–	–
Intermediate	1.64	[0.88–3.08]	0.11
Advanced/expert	3.13	[1.64–5.93]	< *0.001*

According to our analysis, all DSS were significantly associated with increased operative time and with blood losses (Table 2).

Of note, only Kawaguchi and Halls DSS seemed to be significantly associated with an increasing need for unplanned conversion. Failure to achieve the OO was significantly related to all DSS.

In terms of postoperative results, higher difficulty was significantly associated with a longer hospital stay for all DSS but neither Halls nor Iwate scores were able to significantly discriminate increasing length of stay between low-risk and moderate risk classes. Of note, no significant correlation between different DSS with overall and severe complication were found, only the high-risk class of Kawaguchi and Hasegawa score showed a significant correlation with severe complication (respectively OR 5.07, *p* = 0.02, and OR 16.30, *p* = 0.01). The composite measure PO showed that high-risk classes were significantly associated with failure to achieve this outcome in all DSS.

TO was achieved in 66.1% of the patients; univariate analysis showed that a relationship for all four higher classes of DSS, whereas only the Kawaguchi score was able to discriminate between low-risk and moderate risk classes.

### Random forest models

**Fig. 4:**
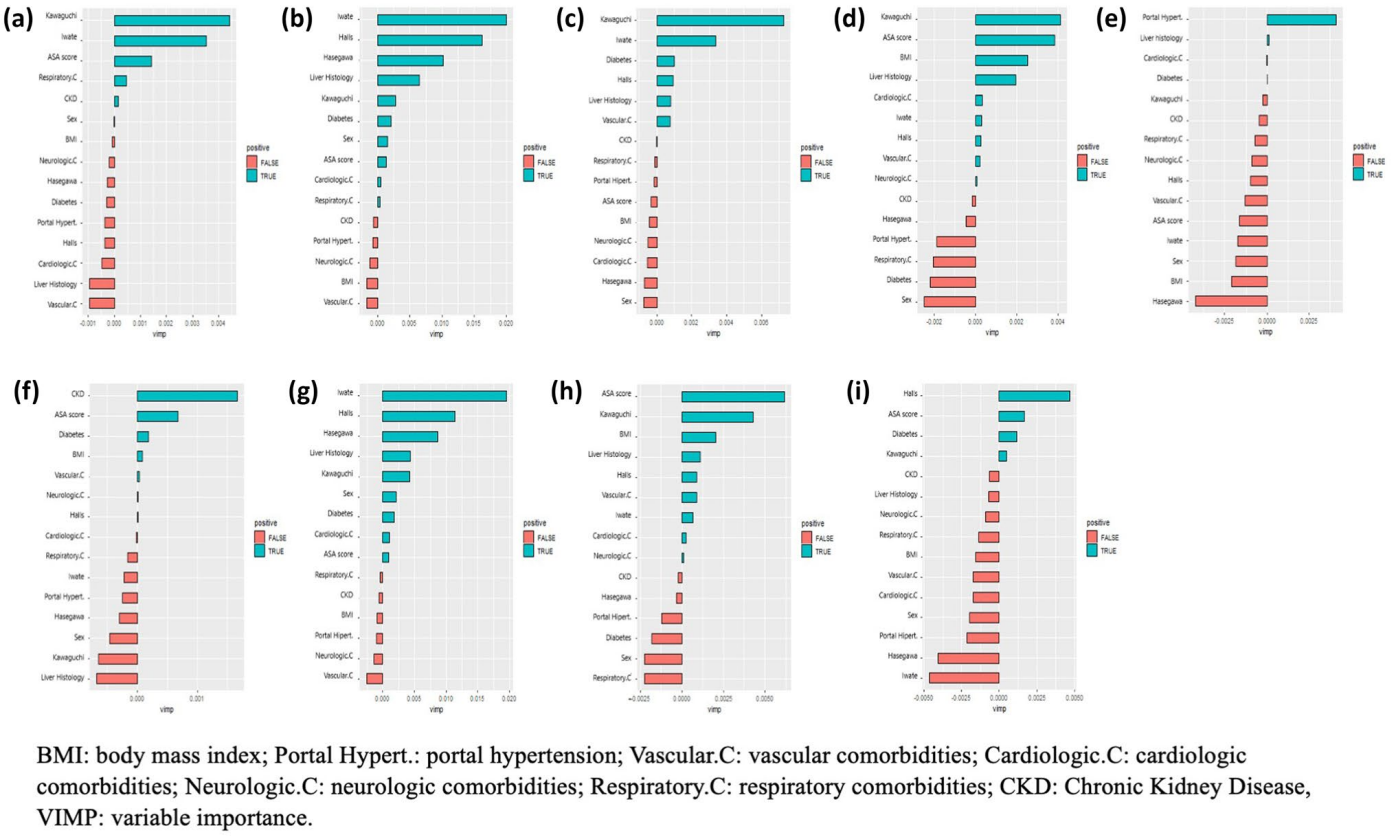
Random forest models designed to investigate the importance of the four DSS in predicting the considered outcomes: **a** excessive blood losses (> 500 ml), **b** prolonged operative time (> 240 min), **c** need for unplanned conversion, **d** prolonged length of stay (> 5 days), **e** incidence of overall complication and **f** severe complications, **g** Operative Outcome, **h** Postoperative Outcome, **i** Textbook Outcome

Random forest models have been implemented to find the most important variable in predicting outcomes, likewise DSS other variables such as BMI, ASA score, sex, liver histology, and specific group of comorbidities (cardiologic, vascular, chronic kidney disease) were included in this machine learning algorithm. The resulting plots are shown in Fig. 4.

In terms of operative outcomes, the random forest analysis showed that all four DSS ranked as the most important variables associated with operative time, with Iwate score as the most important one, followed by Halls, Hasegawa, and Kawaguchi. Analyzing blood losses, we find that Kawaguchi and Iwate were the most important variables associated, followed by the ASA score, while Hasegawa and Halls score seemed to have little importance in predicting blood losses.

Finally, Kawaguchi was the most important variable predicting conversion rate, followed by Iwate and Halls. The composite measure Operative Outcome reflected these results: all the DSS had an important role in predicting it, with Iwate DSS as the most important one.

Considering postoperative outcomes, it can be noted that the Kawaguchi score had the most important role concerning the length of stay, followed by Iwate and Halls, while the Hasegawa score had little to no importance; ASA score, BMI, liver histology, and cardiologic comorbidities had also an important role.

In addition, when analyzing the random forest model for the overall complication, no one of the DSS ranked among the most important variables, whereas the model selected liver histology, presence of portal hypertension, and cardiologic comorbidities.

For severe complications, chronic renal failure, ASA score, diabetes, and BMI ranked as important variables associated, while among DSS only Halls showed little importance.

When considering the composite measure PO, the ASA score had the highest importance, followed by Kawaguchi, Halls, and Iwate scores.

Finally, concerning Textbook Outcome, Halls score, and Kawaguchi score were the only DSS ranking as important variables, together with ASA score and diabetes, in predicting textbook outcome.

## Discussion

In the last decade, there has been a widespread diffusion of mini-invasive liver surgery; the benefits of LLR have been widely proven in literature and consist mainly in reduced blood losses and postoperative pain, length of hospital stay, and complication rates, with oncological results that range from comparable to better than open liver surgery [16, 17].

As a result, many centers implemented a laparoscopic liver surgery program; the first surgeons that developed, standardized, and refined this technique (known as “pioneers”) are now mentoring a new generation of surgeons, the so-called “early adopters” who receive specific training and can faster overcome the learning curve [1].

A key aspect of the learning process is the pre-selection of the cases based on the surgeon’s degree of expertise, meaning which point of the learning curve he reached; as first suggested during the Morioka Consensus Conference, the implementation and the use of difficulty scoring tools may help in this process.

All the considered DSS have been externally validated showing different results; the first one developed, Ban score, later revised, and renewed (Iwate score) has been externally validated by many studies [18–22] finding it useful in predicting technical difficulty and postoperative outcomes. Later on, other DSS have been developed, but they received external validation in fewer studies and did not always perform well: Kawaguchi score was validated on a large cohort and, while depending just on the extent of resection and segments involved, it showed a good correlation with operative time, conversion rate, blood losses, and postoperative morbidity, which was better than the simple major/minor resection distinction and correlated well with Iwate score. Hasegawa score received validation only in two recent studies, in which it was compared with other DSS and performed well in predicting operative and postoperative outcomes. Halls has been externally validated [23], but it has been found lacking correlation with postoperative morbidity or length of stay by Russolillo et al. [24] and had less ability to predict the operative time, the need of Pringle maneuver, and overall morbidity according to Goh et al [25].

In this study, it was decided to test the ability of these four most used DSS to predict 5 individual operative and postoperative outcomes and 3 composite measures, assuming that the more difficult a resection is the higher the incidence of worse outcomes.

The four DSS have been firstly tested for concordance and they showed a low-to-modest correlation: the best concordance was between Kawaguchi and Hasegawa score, followed by Iwate and Kawaguchi and Iwate and Hasegawa, the worst concordance was among those three DSS and Halls score. This finding is consistent with what is reported in the literature and may be related to the fact that Halls DSS incorporates more patient- or disease-related factors and gives them higher weight, while the other DSS are based only or mainly on technical aspects [24].

A logistic regression analysis has been conducted to evaluate the performance of the single DSS in predicting the considered outcomes: the scores performed well in terms of operative time and blood losses, even if Iwate and Kawaguchi scores were not able to highlight differences among their low and moderate difficulty classes; Both Iwate and Hasegawa score, however, showed no statistical significance in terms of predicting conversion. When combining these outcomes in a composite measure called operative outcome (OO), all the DSS highlight statistically significant differences among their classes.

All DSS performed well in predicting prolonged length of stay, even if Halls and Iwate scores were not able to discriminate among low-risk and moderate risk classes. In addition, there was no correlation between the DSS and overall or severe complications, and no discrimination, except for the Hasegawa DSS that was able to discriminate among high-risk and medium risk classes. This was reflected in the results of the scores in predicting PO since all DSS showed a statistically significant difference among risk classes, but Iwate and Halls's scores were not able to discriminate among low-risk and modest risk classes.

In our experience, the TO was reached in 66.1% of the patients, a percentage which is similar to other case series of hepatobiliary surgery [6, 26, 27]; all DSS showed a significant ability to predict whether the TO was reached, but only between high-risk classes and lower difficulty classes; only Kawaguchi score was able to discriminate between low-risk and moderate risk classes.

Given these results that suggested that other factors are implied in determining the considered outcomes in our experience, random forest models were created to investigate the real “importance” of those DSS in predicting the considered outcomes. The best performing DSS according to this analysis was Kawaguchi, that was the most important variable in predicting two out of three operative outcomes (blood losses and conversion) and length of stay, the best performing DSS in predicting the composite PostOperative Outcome, and the only other DSS important in predicting TO with Halls score. Moreover, the Iwate score was the most important in predicting operative time and was among the top-ranking variables also for blood losses, need for conversion, so it is no surprise that it ranked as the most important variable in predicting the composite Operative Outcome.

From the results of this study Kawaguchi and Iwate score, that are based more on technical aspects, predict well the operative outcomes, while textbook outcome, which is a more patient-centered mean of quality evaluation, is better predicted by Halls score, that otherwise performed poorly. Other validation analyses recently conducted suggested that Kawaguchi DSS, given its simplicity and its good results, should be preferred to the others and underline how technical aspects tend to be in the end the most important ones in predicting worse operative and short-term outcomes [25, 28].

One of the most interesting of our results is that throughout all the conducted analysis no DSS showed any correlation with or importance in predicting complications, both overall or severe; the most important variable, among those considered, in predicting complications were liver and patient-related factors such as liver histology, portal hypertension, ASA score, and comorbidities.

While we found out that Kawaguchi DSS has the best performance in our experience, we can say that the technical aspects cannot completely explain the occurrence of complications in our case series, and while they have a notable impact in predicting operative outcome, patient-related factors probably have a more important role in predicting short-term outcomes.

The results of the present study should be considered according to the presence of limitations: the retrospective nature of the study, even if data are collected in a prospectively maintained database. Although the mono-centricity nature of the study is a limitation it should be underlined that standardization of treatment, in patient selection criteria and postoperative management can provide a more accurate evaluation of DSS performance. The strengths of the study are the large number of patients involved in a small period of time and the use of the random forest models, a statistical analysis that tends to limit the “overfitting effect” that is usually imputed to the DSS [2], and underlining the real importance of the DSS in predicting operative and short-term outcomes. Moreover, to the best of our knowledge, this is the first study that investigated how well DSS predict composite outcomes such as textbook outcomes.

## Conclusions

According to our results DDS are significantly related to surgical complexity and short-term outcomes, Kawaguchi and Iwate DSS showed the best performance in predicting operative outcomes; while Halls score was the most important variable in predicting textbook outcome. Interestingly, none of the DSS showed any correlation with or importance in predicting overall and severe postoperative complications.

## Data Availability

The database gathering all data used for this article is available for the Editor of this article for review.

## Abbreviations

DSS: Difficulty scoring systems
LLR: Laparoscopic liver resection
MILS: Mini-invasive liver surgery
BMI: Body mass index
CD: Clavien–Dindo
ASA: American Society of Anaesthesiology
